# Quantitative high resolution melting: two methods to determine SNP allele frequencies from pooled samples

**DOI:** 10.1186/s12863-015-0222-z

**Published:** 2015-06-13

**Authors:** Roxana L. Capper, Young K. Jin, Petra B. Lundgren, Lesa M. Peplow, Mikhail V. Matz, Madeleine J. H. van Oppen

**Affiliations:** Department of Cell and Molecular Biology, University of Texas at Austin, Austin, TX 78712 USA; School of Marine and Tropical Biology, James Cook University, Townsville, Qld 4811 Australia; Australian Institute of Marine Science, PMB3, Townsville MC, Qld 4810 Australia; Department of Anatomy and Developmental Biology, School of Biomedical Sciences, Monash University, Clayton, VIC 3800 Australia

**Keywords:** Pooled genotyping, Allele frequency, High resolution melting, SNP, Population

## Abstract

**Background:**

The advent of next-generation sequencing has brought about an explosion of single nucleotide polymorphism (SNP) data in non-model organisms; however, profiling these SNPs across multiple natural populations still requires substantial time and resources.

**Results:**

Here, we introduce two cost-efficient quantitative High Resolution Melting (qHRM) methods for measuring allele frequencies at known SNP loci in pooled DNA samples: the “peaks” method, which can be applied to large numbers of SNPs, and the “curves” method, which is more labor intensive but also slightly more accurate. Using the reef-building coral *Acropora millepora*, we show that both qHRM methods can recover the allele proportions from mixtures prepared using two or more individuals of known genotype. We further demonstrate advantages of each method over previously published methods; specifically, the “peaks” method can be rapidly scaled to screen several hundred SNPs at once, whereas the “curves” method is better suited for smaller numbers of SNPs.

**Conclusions:**

Compared to genotyping individual samples, these methods can save considerable effort and genotyping costs when relatively few candidate SNPs must be profiled across a large number of populations. One of the main applications of this method could be validation of SNPs of interest identified in population genomic studies.

**Electronic supplementary material:**

The online version of this article (doi:10.1186/s12863-015-0222-z) contains supplementary material, which is available to authorized users.

## Background

Population genomics seeks to link genome-wide genetic variation to evolutionary processes. By querying a large number of individuals for multiple single nucleotide polymorphisms (SNPs) distributed across the genome, polymorphisms with unexpected patterns of genetic differentiation can be identified. These SNPs may be linked to loci under divergent selection between disparate environments [1]. Recent advances in high-throughput sequencing have made it easy to identify large numbers of SNPs in a limited number of individuals [2]. However, profiling a smaller number of SNPs across many populations, for example to study allele frequencies at specific candidate SNP loci, remains time consuming and expensive if based on individual genotyping.

There are many conceptual approaches to determine allele frequencies from pooled samples, including (*i*) direct sequencing [3, 4], (*ii*) primer extension using pyrosequencing [5, 6], SNaPShot [7] or MALDI-TOF mass spectrometry [8], and (*iii*) preferential hybridization or amplification using allele-specific primers, such as TaqMan [9], qPCR [10], the Illumina GoldenGate platform [11], or hybridizing microarrays [12]. For a more extensive review of quantitative genotyping technologies, see Sham et al. and Garvin et al. [13, 14]. Many of these technologies show high correlations between the estimated genotype frequency from the pooled sample and the true allele frequency assessed from genotyping of individual samples, but most require considerable investment of funds and effort for each new SNP assay. While direct sequencing with next-generation sequencing methods is an increasingly popular option, the sequencing of whole genomes, reduced representation libraries (RRL), or even specific amplicons incurs unnecessary costs for research that seeks to investigate the frequencies of a subset of preselected candidate or otherwise potentially informative (“tag” or “proxy”) SNPs across populations.

Conventional HRM has been established as a very sensitive tool for detecting even low levels of a target allele [15]. Quantitative HRM methods have been developed in a range of applications [16], including detection of the adulteration of food or drug products [17–20], methylation status [21–23] and species composition in samples over time [24]. One commonality of these approaches is that they make use of relatively large differences among variants, such as multiple methylated sites within an amplicon, multiple SNP differences among species in the locus of interest or even entirely different genes representing different species. These methods are not sufficiently sensitive to detect frequencies of single SNPs among populations of the same species.

The conventional, unlabeled probe version of high resolution melting (HRM) method of SNP genotyping uses a small oligonucleotide probe to increase the differences in melting temperature between single SNP alleles. It makes use of a dye that fluoresces only when intercalated into duplex DNA structures. First, the SNP-bearing locus of interest is amplified with asymmetric PCR such that an excess of one strand is produced. Next, the short probe that overlays the SNP site itself is added to the reaction. The reaction is subsequently heated, cooled and heated again while the loss of fluorescent signal (i.e. duplex DNA disassociation) is monitored. The melting temperature of the probe-SNP duplex region is dependent on the SNP allele state; a perfect match will have a higher melting temperature than will a mismatch [25]. A conventional HRM assay typically seeks to determine whether the probe melting profile contains only the higher-temperature component, the lower-temperature component, or both.

In this study, we explore whether the unlabeled probe HRM melting profile can provide quantitative information about frequencies of alternative alleles in a pooled DNA sample. We developed two versions of qHRM that use different dye chemistries, brands of HRM machines and analytical techniques to quantify the relative proportions of each allele. We performed a total of six experiments involving adult *Acropora millepora* corals, an emerging ecological genomics model, to demonstrate the new method’s accuracy.

## Methods

### Coral collection

Adult corals were used in all experiments. For the allele titration and SNP panel validation experiments (peaks method), corals were collected from Trunk Reef (−18.368 S, 146.818 E) and Little Pioneer Bay, Orpheus Island (−18.604 S, 146.486 E), Great Barrier Reef, Queensland, Australia in 2009. Samples used in the Orpheus/Magnetic population comparison experiment (peaks method) were collected from Orpheus Island and Nelly Bay, Magnetic Island (−19.165 S, 146.851 E). Coral samples used to validate the curves method experiments were collected from 28 coral populations, summarized in Additional file 1. All corals were collected under the appropriate Great Barrier Reef Marine Park Authority permits.

### DNA extraction

For the coral individuals used in the allele titration and SNP panel validation experiments (peaks method), genomic DNA was extracted using the Qiagen DNeasy Blood and Tissue kit (Qiagen) and was eluted into Buffer EB. Individuals sampled from Orpheus and Magnetic Islands (peaks method) and from the 28 populations across the Great Barrier Reef (curves method) were extracted according to the method described in Wilson et al. [26]. DNA concentrations were initially determined using the Nanodrop ND-1000 Spectrophotometer (Thermo Scientific).

### Peaks method

**Fig. 1:**
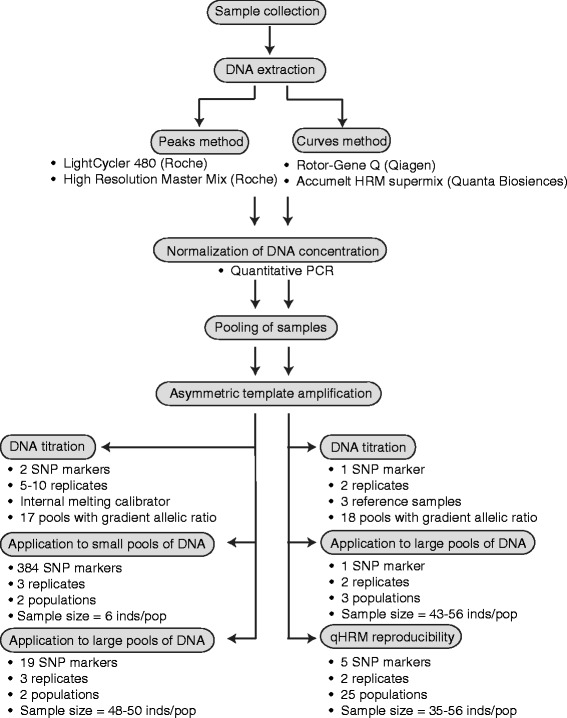
Flow chart of qHRM, including benchmarking experiments performed for each method

The peaks method was developed as a rapid means to quantify SNP allele frequencies across several hundred SNP loci. Briefly, we first established proof of concept using titrated allele frequencies and two SNP markers, then validated a panel of 384 SNPs using six corals, genotyped individually and as a pool. This panel of SNPs was finally applied to samples of two natural coral populations to detect loci with different frequencies between the reefs. The workflow of the peaks method is illustrated in Fig. 1.

### Pooling

In order to convert conventional HRM into a quantitative assay for pooled samples, equal amounts of each individual’s DNA must be added to the initial asymmetric PCR reaction. This DNA normalization step is particularly critical for pooling organisms that may have known or unknown assemblages, infections or symbioses as the relative amounts of target and contaminating DNA can vary among samples. To circumvent these problems, we used quantitative PCR (qPCR) to accurately measure the quantity of coral DNA in individual samples. Primers to amplify the SNP locus C23209S177 [27] were used in a conventional qPCR reaction performed under the following conditions using a LightCycler 480 machine (Roche): 5 ng holobiont DNA, 0.1 μM each forward and reverse primers, 2 mM MgCl_2_ and 1x High Resolution Master Mix (Roche) in a 15 μl volume. Reactions were heated to 95 °C for 10 minutes, then cycled 55 times as follows: 40 s at 95 °C, 40 s at 60 °C, 40 s at 72 °C, then cooled to 40 °C and held for 20s. Concentrations of target DNA were normalized based on differences in quantification cycle (Cq) by assuming that the fold-difference per each Cq unit difference was equal to 1/*E*, where *E* is the amplification factor per PCR cycle for the particular primer set [28]. The accuracy of this approach was verified by re-amplifying the adjusted DNA concentrations again with the same primer pair.

### Internal melting calibrators

**Fig. 2:**
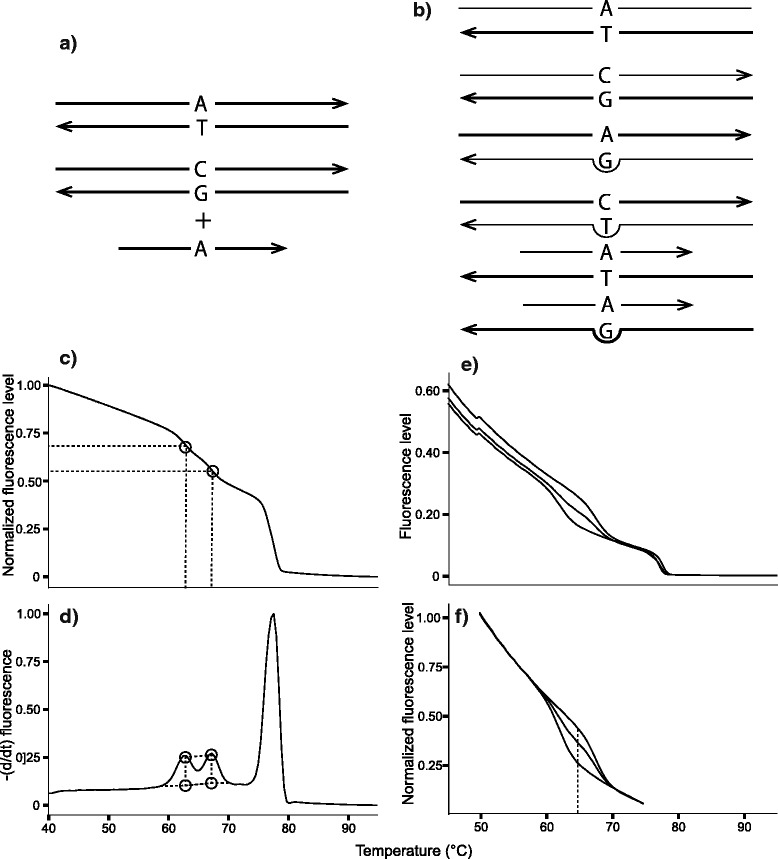
Schematic diagram of qHRM. An individual heterozygous for a particular SNP locus is shown as an example. **a** For both methods, the locus is amplified from genomic DNA using asymmetric PCR to generate an excess of the reverse strand. Next, an oligonucleotide probe is added to the reaction. **b** The mixture is heated to melt all duplexes apart, and subsequently cooled rapidly to promote unbiased duplex reformation of all possible duplex DNA species. For a biallelic SNP site, this produces six distinct types of duplexes: two perfect-match amplicon duplexes, two mismatch amplicon duplexes, one perfect-match probe duplex, and one mismatch probe duplex. **c** For the peaks method, the reaction is heated again to denature all duplexes while the fluorescence of the reaction, which quantifies double stranded DNA, is monitored. **d** Next, the negative first derivative of the decreasing fluorescence curve is calculated to transform the curve into a peaks profile. The height of the lowest-melting, mismatched probe duplex peak is divided by the total heights of both probe peaks to yield the frequency of the mismatched allele in the sample. **e** For the curves method, the same process was repeated as described for panel c. Three known samples that consist of each genotype (i.e. heterozygote, high-melting homozygote and low-melting homozygote) were used as references. **f** Melt curves from the raw channel were normalized by averaging fluorescence value outside a melting phase and forcing the values to be the same for each sample

Though within-plate variation among replicates was low for the peaks method, we noticed that between-plate variation could shift the melting profile of all duplex DNA species (i.e. for an analyzed heterozygote, such species include two perfect-match amplicons, two mismatched amplicons, one perfect-match probe-SNP duplex, and one mismatch probe-SNP duplex; Fig. 2a,b) by as much as 2 °C. To accommodate this phenomenon, which occurred only in the peaks method, we included standard internal melting calibrator primers with each reaction in order to provide landmarks in the melting profile. Four oligonucleotide calibrators were designed to form two complementary DNA duplexes with known melting temperatures. They were based on sequences from Gundry et al. [29] but were adjusted to melt at the slightly lower and higher temperatures of 47.5 °C and 90 °C than the published sequences in order to melt well outside of the range of the probe and amplicon duplexes. The 3′ terminal ends of each of the four oligos were modified with an inverted T to block any potential primer extension from occurring. The forward sequence of the low-melting calibrator used is 5′- ATT TTA TAT TTA TAT ATT TAT ATA TTT TT/3InvdT/ -3′, while the forward sequence of the high-melting calibrator is 5′- GCG CGG CCG GCA CTG ACC CGA GAC TCT GAG CGG CTG CTG GAG GTG CGG AAG CGG AGG GGC GGG/3InvdT/. The calibrator oligos were included in the initial asymmetric PCR reaction at a concentration of 0.05 μM for each of the high-melting oligos and 0.5 μM for the low-melting oligos; addition of the calibrators did not interfere with amplification of the target products.

### Asymmetric template amplification and melt stage

In HRM, the asymmetric PCR stage serves to generate an excess of the amplicon strand that is complementary to the probe. This excess strand can then form a duplex with the unlabeled probe, whereas if it was not in excess it would preferentially bind to the longer, complementary amplicon strand instead. Though some versions of HRM use symmetric PCR, their applications typically involve whole-amplicon melting analysis. In contrast, unlabeled probe HRM, which yields the increased sensitivity necessary for SNP discrimination, requires asymmetric PCR in order to outcompete the amplicon. The asymmetric PCR reaction prior to each assay’s melt stage was performed as follows on the LightCycler 480 (Roche): 5 ng total pooled DNA, 0.3 μM reverse primer, 0.067 μM forward primer, 0.05 μM each high-melting calibrator forward and reverse oligos, 0.5 μM each low-melting calibrator forward and reverse oligos, 2 mM MgCl_2_, and 1x High Resolution Master Mix (Roche). Reactions were heated to 95 °C and held for 10 m, then cycled as follows: 40 s at 95 °C, 40 s at 60 °C, 40 s at 72 °C for 55 cycles, which, for most SNPs, was at least 10 cycles past the beginning of the reaction plateau. The reaction was then finally cooled to 40 °C and held for 20 s.

Following the asymmetric target amplification, the reaction plates were unsealed and 0.5 μM of the oligo probe complementary to the excess reverse strand of the amplicon was added. The plates were resealed and then heated to 95 °C for one minute to melt all double-stranded DNA species apart. Subsequently, the plates were rapidly cooled to 45 °C and held for one minute to allow reannealing of all possible probe and amplicon duplexes. Next, the reactions were heated to 95 °C at the maximum rate allowed by the Roche LightCycler 480 (>0.02 ° C/s). During this heating period, the LightCycler 480 monitored the amount of fluorescence over time and collected over 1300 data points over a 50 °C range, producing a high-resolution graph of the melting profile of each species of duplex DNA in the reaction.

### Analysis of denaturation profiles

We calculated the negative first derivative of the melt stage’s decreasing fluorescence curve to produce a profile with discrete peaks centered at the melting temperature of each dissociating duplex (Fig. 2c,d). Next, we drew a baseline connecting the linear regions bounding the melting peaks to determine the background rate of dye disassociation. Then, the height of the probe melting peaks above the baseline was measured using a custom [R] script (see Additional file 2). For pooled reactions, three technical replicates were performed for each locus and sample pair and their relative peak heights averaged. The genotype of a sample was determined by the presence and position of the probe peaks, relative to other samples for the same locus and to the calibrator melting peaks. The frequency of the low-melting allele in a pooled sample was estimated by the contribution of its corresponding melting peak to the total summed height of probe-related melting peaks. Across all SNP assays, a minor allele was called only if it exceeded a frequency of 2 %.

### DNA titrations

To demonstrate a linear relationship between known frequencies of a given allele and the qHRM-estimated frequency estimated from a pooled sample, we first identified two individuals homozygous for different alleles of the same SNP. We performed this experiment for two SNP loci mined from the *A. millepora* transcriptome, C22162S248 and C45133S676 [27]. We normalized the concentrations of DNA with qPCR as described above, then mixed the two homozygotes in different proportions to obtain mixtures that incremented the frequency of the low-melting allele from 0–100 % in 5 % or 10 % steps (Additional file 3). qHRM was then performed on each mixture with five replicates for each mixture for two independent normalization and mixing trials, for both SNPs assayed. The estimated allele frequencies for all replicates of each mixture were then averaged and compared to the known allele frequencies.

### Analysis of 384 loci in six individuals

DNA samples from three adult coral individuals sampled from Trunk Reef and three from Orpheus Island were genotyped individually by conventional HRM for 384 loci selected from SNPs previously developed for a previously published coral linkage mapping project [27] (Additional file 4). Following this, the samples were normalized for coral DNA concentration as explained above and pooled to generate a DNA sample with known allele frequencies for each SNP locus. qHRM was performed in triplicate on the pooled sample for the same 384 SNPs. To fully validate qHRM of each SNP in this panel, we only analyzed data from SNPs for which all six individuals were both successfully amplified and had easily distinguishable peaks when analyzed using conventional HRM, and which had clear qHRM peaks greater than 2 % of the total fluorescence signal in the reaction (usually relative to the amplicon peak’s height). At least two successful replicates were required for each SNP in the qHRM reactions for analysis to proceed.

### Analysis of 384 loci in 98 individuals collected from two locations

We used the peaks method to compare allele frequencies in two populations of corals collected from Magnetic Island (*n* = 48) and Orpheus Island (*n* = 50). DNA was normalized, pooled according to reef of origin, and qHRM-genotyped in triplicate using a panel of 384 SNPs. Nineteen of these SNPs were further validated by individual HRM genotyping of each individual contributing to the pools. The set of SNPs to validate was selected to represent loci where the low-melting allele was major (8 SNPs) or minor (11 SNPs), loci with small or large minor allele frequencies (11 SNPs with MAF < 0.25; 8 SNPs with MAF > 0.25), SNPs with more than two alleles (7 SNPs with third alleles in one or both populations), and loci selected to span the range of estimated F_ST_ values (7 SNPs with F_ST_ less than 0.01; 4 SNPs with F_ST_ between 0.01 and 0.02; 8 SNPs with F_ST_ greater than 0.02, including four of the top seven SNPs with the greatest F_ST_).

### Analysis of genetic subdivision between coral populations

SNP allele frequencies were compared between Orpheus and Magnetic populations by plotting the averaged qHRM-estimated frequencies against each reef.

F_ST_ for each SNP was calculated according to Equ. 1, where *p* is the frequency of the high-melting allele, *q* is equal to (1-*p*), $\overline{p}$ is the average frequency of *p* allele between the two populations, and $\overline{q}$ is equal to $\left(1-\overline{p}\right)$.

1 $$ {\mathrm{F}}_{\mathrm{ST}}={\frac{\left({p}_1-{p}_2\right)}{4\overline{p}\overline{q}}}^2 $$

Global F_ST_ was calculated by averaging all SNP F_ST_ values.

### Curves method

The curves method was developed to extend qHRM and demonstrate functionality across another dye chemistry and analytical approach. This method is best suited for highly accurate determination of allele proportions across small numbers of SNPs. It requires the usage of three reference sequences (two homozygous samples and a heterozygous sample) for comparison with an unknown, pooled sample. We demonstrate that reactions are extremely reproducible and robust among different markers and pooled samples. The workflow of the curves method is illustrated in Fig. 2. Briefly, the curves method was validated through an initial proof of concept experiment using different known proportions of alleles for a single SNP marker. This marker was additionally validated on three DNA pools made from 43–56 individuals representing distinct coral populations by comparing the true allelic frequency of the pool (revealed by summing the genotypes of each individual) to the qHRM-estimated frequency. Thirdly, reproducibility of this method was investigated by using five SNP markers for pooled samples representing 25 coral populations.

### Pooling

Each individual sample was amplified individually in an asymmetric qPCR reaction using primers for the locus C70S236 [27] under the following conditions using a Rotor-Gene Q machine (Qiagen): 0.1 μM forward primer, 1 μM reverse primer, 10 ng holobiont DNA, 1× 7.5 μl of the Accumelt HRM supermix (Quanta Biosciences) in a 15 μl volume. The reaction mixture was heated at 95 °C for 10 min, then cycled 40 times with the following thermoprofile: 95 °C for 40 s, 58 °C for 40 s, and 72 °C for 40 s. DNA concentrations were normalized according to Pfaffl [28] as summarized in the Peaks - pooling section.

### Asymmetric template amplification and melt stage

After normalization of individual samples, each individual was combined into a single pooled sample. The pooled sample was then was amplified asymmetrically with the Rotor-Gene Q machine (Qiagen) with the locus C70S236. Next, 1 μM of probe complementary to the excess strand was added to each reaction. Then, the reactions were heated to 95 °C and held for 60 s, rapidly cooled to 45 °C and held for 150 s, heated again to 95 °C at a rate of 0.1 °C/s with a 2 s hold each step, collecting 500 data points in total.

### Analysis of denaturation profiles

Because many factors such as pipetting errors causing variations in quantity of fluorescent dye and DNA can affect the relative fluorescence levels among normalized DNA samples, it is important to standardize the fluorescence levels to exclude the noise for accurate allele frequency measurements. Fluorescence values of the SNP-specific melt curves were standardized by selecting regions before and after the probe melting phase where nucleotide differences do not lead to variations in fluorescence level, then averaging fluorescence levels of the selected regions among all samples using the Rotor-Gene Q Series Software 2.0.2.4 (Corbett) in order to mitigate the effects of sample- and SNP-specific variation (Fig. 2e). The exclusion of noise through this normalization process allows the direct comparison of melting curves from different reactions.

The inclusion of three standard reference samples of known genotype (two different homozygotes and a heterozygote) allows for even small differences in allele frequency to be resolved. These standards set a reference fluorescence level to which unknown samples can be compared for absolute allele frequency quantification (Fig. 2f) and also serve to reduce variations between runs.

The y-axis point at which the greatest difference in fluorescence between the two homozygotes occurs is used to determine the relative proportion of alleles in an unknown sample as it gives the best highest accuracy in estimating allele frequencies (Additional file 5). This point should also be near where the inflection point of the heterozygote’s curve falls. But, because the heterozygote can suffer from amplification biases, the position of the inflection point can vary. We see increased gains in accuracy by calculating the allele frequency of the unknown sample with respect to the heterozygote rather than simply using the midpoint of the two homozygotes. For example, if an unknown sample has a higher frequency of the high-melting allele (i.e. the unknown’s curve falls above of the heterozygote’s curve and/or the point of the greatest distance between homozygotes), then Equ. 2 can be used to estimate of the proportion of the high-melting allele. If the unknown has a higher frequency of the low-melting allele (i.e. the unknown’s curve falls below the heterozygote’s curve and/or the point of the greatest distance between homozygotes), then Equ. 3 should be used to calculate the frequency of the high-melting allele. In both equations, we calculate the proportion of fluorescence at the inflection point of the unknown sample (*x*) relative to the heterozygote (*f*_*het*_*)*. After adjusting for empirical differences between the known heterozygote and one of the known homozygotes (*f*_*high*_ or *f*_*low*_), we add or subtract the value from 0.5 to determine the allele frequency of the unknown sample relative to the low-melting homozygote.

2 $$ \mathrm{frequency}\kern0.5em \mathrm{of}\kern0.5em \mathrm{high}\hbox{-} \mathrm{melting}\kern0.5em \mathrm{allele}=0.5+0.5\left(\frac{x-{f}_{het}}{f_{high}-{f}_{het}}\right) $$

3 $$ \mathrm{frequency}\kern0.5em \mathrm{of}\kern0.5em \mathrm{high}\hbox{-} \mathrm{melting}\kern0.5em \mathrm{allele}=0.5-0.5\left(\frac{f_{het}-x}{f_{het}-{f}_{low}}\right) $$

### DNA titrations and method validation

The use of three reference genotypes per SNP assay and the fluorescence standardization step should theoretically allow for great sensitivity to detect allele frequency differences among pooled samples. To assess this, we first identified multiple individuals of each of the three possible genotypes for the locus C70S236 and normalized DNA concentrations of each sample. We then combined them into one pool per genotype and mixed them in varying proportions to generate a gradient of alleles spanning 0–100 % of the low-melting allele by 5–10 % increments. The estimated allele frequencies for two technical replicates of each mixture were averaged and compared to the expected allele frequencies (Additional file 3).

### Application to large pools of DNA

DNA sampled from individual corals sourced from Myrmidon (*n* = 43), Night (*n* = 56) and Wilkie (*n* = 49) Reefs was normalized and individually genotyped using SNP C70S236. Normalized samples were then pooled by population and quantitatively genotyped in duplicate in order to examine the accuracy of the curves method when used with many individuals per pool.

### qHRM reproducibility

Next, we evaluated the reproducibility of qHRM on 25 populations sampled along the Great Barrier Reef. We pooled DNA from 35–56 individuals for each population, and then performed curves qHRM on each pool in duplicate for five SNPs (C29226S281, C70S236, C11461S560, C16774S791 and C20479S292), which were all individually normalized as per above.

Consistency between the two replicates was measured by estimating the half-confidence interval of the difference as follows:

$$ \frac{z\times SD}{mean} $$

Standard deviations (SD) and mean values were calculated using fluorescence levels in the temperature range where the largest difference in fluorescence levels between the two homozygotes were observed (Fig. 2f). A z value of 1.96 was used so the measure of precision represents half of a 95 % confidence interval.

We further examined the qHRM reproducibility for the 5 markers and 25 reefs by comparing mean errors. Errors in percentage were calculated as follows:

$$ \mathrm{Percentage}\kern0.5em \mathrm{of}\kern0.5em \mathrm{error}\kern0.5em \mathrm{between}\kern0.5em \mathrm{replicates}=\left(\frac{f_1-{f}_2}{\left({f}_1+{f}_2\right)/2}\right) $$

where *f*_1_ and *f*_2_ are two replicates of fluorescence data. Geometric means of errors were used as population- and marker-specific errors.

## Results

### Peaks method

#### Allele titrations

**Fig. 3:**
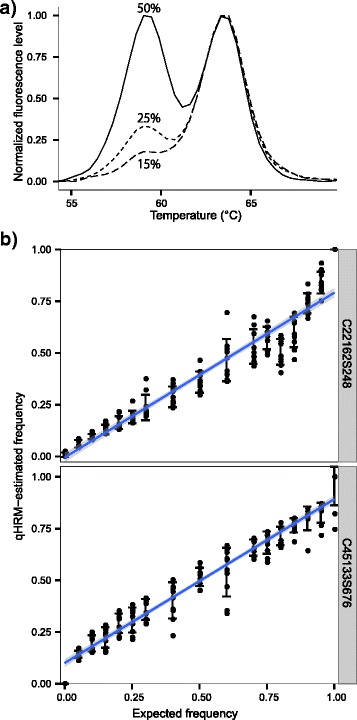
DNA titrations using the peaks method. **a**) Probe melting peaks for different allele titrations demonstrating clear resolution among allele frequencies (15 %, 25 %, 50 %). **b**) Two homozygotes were mixed in varying proportions to test qHRM using SNP C45133S676 (Pearson’s r = 0.97, regression slope = 0.82) and SNP C22162S248 (Pearson’s r = 0.99, regression slope = 0.80). The qHRM estimates were tightly correlated to expected frequencies; however, qHRM appears to underestimate the true allele frequency for the low-melting allele for both SNPs considered.

As an initial proof of concept, we analyzed DNA from two adult individuals determined to be homozygous for different alleles of the same SNP, mixed in varying proportions to represent a spectrum of allele frequencies. The probe peaks for three allele frequency examples (50 %, 25 %, 15 %) are presented in Fig. 3a. For the two SNPs analyzed in this way, there was a strong linear correlation between the qHRM estimations and the true proportion (Pearson *r* = 0.97-0.99). However, the slope of the regression for both SNPs was 0.80 (Fig. 3b).

#### Analysis of 384 loci in six individuals

**Fig. 4:**
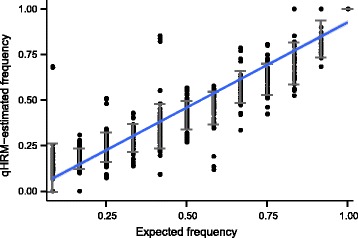
Analysis of 384 loci in six individuals (Peaks method). Six adult corals were HRM-genotyped individually for 384 SNPs, then qHRM-genotyped as a pool in triplicate. The expected allele frequency was calculated by summing the genotypes for each individual (12 alleles possible), and the qHRM-estimated frequencies were calculated as described in the Materials and Methods section. The Pearson correlation between the expected and the qHRM-estimated values is 0.89. The slope of the linear regression is 0.94

To further validate the method, DNA samples from six adult coral colonies from two reefs were individually genotyped for 384 SNPs selected from the coral linkage map using conventional HRM. DNA from these six individuals was pooled and the allele frequency at each SNP was estimated using qHRM. Of the 291 SNPs that passed quality filtering, 54 were homozygous, all of which were correctly identified by qHRM. For 237 polymorphic SNPs, the Pearson correlation between the expected and estimated allele frequencies was 0.89 with a regression slope of 0.94 (Fig. 4), qHRM failed to detect polymorphism for eight of the polymorphic SNPs, all of which had minor allele frequencies of 8 %, the lowest possible allele frequency in this experiment.

#### Analysis of 384 loci in 98 individuals collected from two locations

To demonstrate that qHRM can identify SNPs with different allele frequencies among real populations, we surveyed the same 384 SNPs in pooled samples representing the Magnetic Island and Orpheus Island populations of *A. millepora*. These populations are geographically close together but have very different environmental parameters and some degree of genetic separation (F_ST_ = 0.041) [30].

**Fig. 5:**
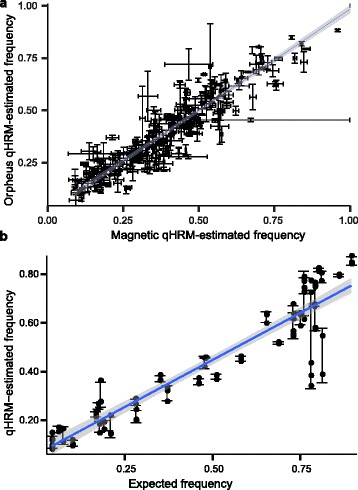
Analysis of 384 loci in 98 individuals collected from Orpheus Island and Magnetic Island. **a** 48 and 50 individuals from the respective reefs were pooled and quantitatively genotyped using qHRM. The allele frequencies between the reefs were tightly conserved between the two populations, with a Pearson’s *r* of 0.93 and a slope of 0.97. **b** We further validated 19 SNPs by genotyping individual members of each pool. The Pearson correlation between known and pooled genotypes for these SNPs is 0.93 with a regression slope of 0.77

Of the 384 SNPs assayed, 261 were represented by at least two technical replicates in each population, including 34 monomorphic loci. Notably, the qHRM-estimated allele frequencies for the 261 loci were very similar among populations (Pearson’s *r* = 0.93, slope = 0.97) (Fig. 5a). Global F_ST_ between the Magnetic and Orpheus populations using 261 SNPs was calculated as 0.006.

We further validated the qHRM allele frequency calls for 19 SNPs by individually genotyping members of each population (total *n* = 98) using conventional HRM. The Pearson correlation between expected allele frequencies and the qHRM-estimated frequencies for these 19 SNPs was 0.93 with a regression slope of 0.77 (Fig. 5b). Three of the 19 SNPs from Orpheus Island and two from Magnetic Island populations failed to generate reliable HRM data for one to six individuals, suggestive of a mutation in the HRM primer biding site(s), and therefore were not possible to fully validate. However, it is likely that these missing individuals also failed to contribute to the pooled sample’s allele frequency estimate.

#### Detection of novel alleles

Unlike other technologies that are only able to detect the alleles sought and may therefore suffer from ascertainment bias, HRM is able to detect novel sequence variants within the probe-SNP duplex, which manifest as additional melting peaks. During the course of this study, qHRM suggested the presence of a third SNP within the interrogated probe region in 34 (9 %) of SNP assays tested for the pool of six individuals collected from Trunk Reef and Orpheus Island. In another assay, we found four distinct melting peaks across six separately HRM-genotyped individuals with a maximum of two alleles for each sample, though a separate SNP assay revealed three alleles within a single sample. This could indicate a high level of polymorphism (multiple SNP sites within the 20-base probe region), a potential genomic duplication event or a chimeric individual (Additional file 6). Each unusual case was validated by individual HRM genotyping.

Additionally, for the analysis of the Orpheus Island and Magnetic Island populations, we identified third alleles in qHRM for 9 SNP assays in the Magnetic Island pooled sample and for 10 SNP assays in the Orpheus Island pooled sample. Of the 19 assays included in the individual validation experiment, 7 revealed three alleles in one or both populations. For several of these assays, the third allele was not detected by qHRM because its frequency was too low or was otherwise undetectable in the pooled sample.

### Curves method

#### Allele titrations

**Fig. 6:**
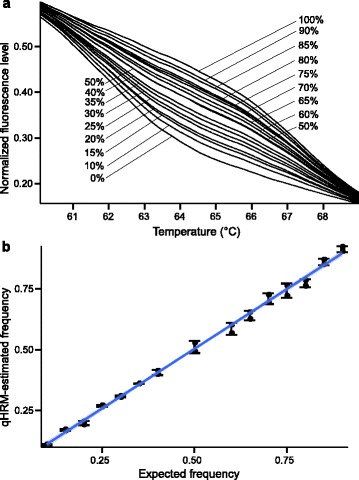
DNA titrations using the curves method. **a** HRM normalized curves produced from sample batches that were mixed at gradient allelic ratios. **b** A linear regression showing a relationship between estimated and expected low-melting allele frequencies of the C70S236 locus. Pearson’s *r* = 1. Slope=0.982

As with the peaks method, the curves method showed a high correlation between true and estimated allele frequencies with only two replicates using the SNP marker C70S236 (Pearson’s *r* = 1 and slope = 0.982) (Fig. 3). Furthermore, error rates ranged between 0.0017 and 0.026 with a standard deviation of 0.006, which verifies the high accuracy of the curves method for absolute quantification of allele frequencies with this marker (Fig. 6a,b).

#### Application to large pools of DNA

The curves qHRM method proved highly accurate on pooled DNA samples. The allele frequencies of Myrmidon, Night and Wilkie populations were estimated by both individual HRM and pooled qHRM genotyping using the marker C70S236. There was very close agreement between the true allele frequency, as calculated by summing the allele frequencies for each individual contributing to each pool, and the qHRM-estimated frequency. The differences between estimated allele frequency and the true values were 0.9 % for Night, 1.1 % for Myrmidon, and 3.6 % for Wilkie Reef. An outlier sample was identified in Wilkie population (Additional file 7).

#### Reproducibility of qHRM

Our methods demonstrated high precision in measuring fluorescence levels between replicates (<2 % of Half-CI with mean errors of <0.4 %) for the five markers examined with this method (Additional file 8). Population-specific mean errors for all populations ranged between 0.1 and 0.75 %. A simple calculation using average fluorescence values of the two homozygote reference samples shows that 1 % of fluorescence error is equivalent to about 0.14 % allele frequency error for C70S236.

## Discussion

Genotyping using next-generation sequencing, although no doubt powerful and versatile, is neither feasible nor necessary for applications that must maximize the number of genotyped populations rather than the number of genotyped loci. For example, genetic connectivity studies might not require more than a hundred SNP markers [31], but would instead benefit from careful selection of the markers to ensure that they are physically unlinked, adaptively neutral and polymorphic across the geographical range of interest [32]. Determining their allele frequencies across a large number of distinct populations, on the other hand, can be uniquely beneficial for identifying physical and ecological barriers to migration and disentangling the effects of geographic and ecological isolation on genetic differentiation [33]. Profiling allele frequencies at a few SNPs across numerous populations can be an efficient approach to validate potential targets of natural selection (“outlier” SNPs) suggested by genome scans [32, 34]. Other potential applications for targeted, medium-throughput assays include fine-scale investigations into a specific chromosomal region, profiling a single “tag” SNP for markers that are in high linkage disequilibrium with each other, or metagenomic analysis using a set of markers in order to disentangle species composition.

One of the main hurdles of these approaches, particularly for population or ecological genomic studies in non-model organisms, is overcoming the difficulty and expense of genotyping a sufficient number of individuals to accurately estimate allele frequencies of a population. Some technologies are able to overcome this barrier by using DNA pooling methods in which many individuals can be combined into a single reaction for each group, drastically improving the cost and time efficiency of a study [13]. However, not all genotyping technologies are quantitative and therefore may not be appropriate for use with pooled samples.

Other quantitative applications of HRM have been described [16–24], but to date these methods have been designed to detect relatively dramatic differences in sequence between alleles. These methods have not been successfully applied to the unlabeled probe method of qHRM, where differences between single SNPs are magnified through the use of a small oligo probe. In addition, unlike other qHRM methods that use duplex PCR to amplify different loci or different alleles [35–37], our method should not suffer from biases due to different efficiencies of PCR primers’ binding to alternative alleles because the interrogated SNP is in the middle of the amplicon.

Various quantitative comparison methods that analyze the relative contribution of each variant in a pooled sample have been designed, including determining the amount of fluorescence at an allele’s melting curve inflection point [20], subtracting the unknown sample’s melting curve from a homozygous reference’s melting curve and comparing the heights of differential curve’s peaks [22], or deriving the melting curve into a melting peaks graph and analyzing the areas under the resulting peaks [24]. However, these methods are not directly applicable to our unlabeled probe qHRM method. Because of error introduced when normalizing the probe region subset of a melting curve due to the baseline disassociation of the dye, as well as the necessity to temperature-shift assays across different plates using the internal calibrator sequences, comparing the fluorescence level at an inflection point is difficult. Additionally, both this inflection-point comparison method and the subtractive method require the usage of reference samples, which scales poorly when using more than a few SNP markers. The melting peaks’ area analysis presented in Lin and Gänzle [24] is sophisticated, but it requires the use of proprietary software, deconvolution of peak curves and manual peak modeling. Though accurate, the expensive software and the time involved in calculating peak areas may limit the application of this type of analysis for medium-throughput projects.

Pearson’s correlations between expected allele frequencies and estimated allele frequencies were high for all experiments. The regression slopes of the experiments validated via individual genotyping (allele titration, two SNPs; six individuals, 291 SNPs; and reef comparison, 19 SNPs validated for members of each population) ranged from 0.77 to 0.98. The peaks qHRM method slightly underestimates the true allele frequency of the low-melting allele (probe-mismatch allele). Ostensibly, this could be due to a bias during probe-DNA duplex formation during the initial cooling phase of HRM that favors the perfectly-matching probe. Regardless of its cause, this minor bias is unlikely to affect the conclusions of genetic studies outlined in the beginning of this Discussion since such studies rely upon variation in allele frequency among populations rather than its mean. In this regard, it was particularly encouraging to see very similar allele frequencies as estimated by qHRM between two natural coral populations (validated by individual HRM genotyping), indicating that the peaks method is highly reproducible.

The high reliability of the curves method (Pearson’s *r* = 1 and slope = 0.982) is attributed to the inclusion of heterozygote as reference sample and identification of temperature region that gives the least error in allele frequency estimation. However, since the curves method of qHRM analysis uses reference genotypes to determine the relative contribution of each allele in an unknown sample, an allele that is not represented by the reference genotypes will be missed as a fluorescence produced by a third allele would be merged and difficult to detect by fluorescence of other alleles when pooled.

An alternative to counteracting the effects of the rare alleles and cross-contamination on allele frequency estimation is to increase sample size thereby reduce errors caused by a minority of samples. Johnston et al. [38] showed improved mean adjusted R^2^ and mean difference between estimated and true allele frequencies with increasing numbers of samples (also see Neve et al. [39]).

The low level of sample-specific errors in the curves method proves that the replicate accuracy was not significantly affected by differences in pipetting errors and DNA quality among samples. The accurate replication among samples of different DNA qualities can be attribute to a short length of PCR product (about 100 base pairs) [40].

Gruber et al. [41] demonstrated comparable accuracy and reproducibility of a pyrosequencing technique to the qHRM method presented in this study, but the accuracy of pyrosequencing was compromised in SNPs that were flanked by identical bases to the SNP alleles. In contrast, our qHRM method (the curves method) showed a clear difference in fluorescence level among genotypes for three SNPs (C11461S560, C16774S791 and C20479S292) despite the presence of identical flanking sequences (Additional file 9). This simplifies primer design and eliminates the need for sequencing SNP sites for primer optimization. High accuracy and reproducibility of allele frequency calls for all the 5 SNPs tested for the curves method support its applicability to other biallelic markers.

## Conclusions

The proposed qHRM methods demonstrated high accuracy and reproducibility in several cross-validation experiments. The key advantages of these methods over other targeted allele frequency profiling techniques include the ease of assay design and low overall cost of the analysis. All of these factors make qHRM a method of choice for cases requiring genotyping a few hundred SNP markers across a large number of populations.

### Availability of supporting data

The data sets supporting the article are included within the article and its additional files.

## Additional files

Additional file 1:
**Collection year, sample size, latitudes and longitudes of 28 populations.**
Additional file 2:
**R scripts for peak height finder.**
Additional file 3:
**Known frequency mixtures and their qHRM-estimated frequencies of low-melting allele.** Sample batches that were pooled at gradient allelic ratios. Additional file 4:
**384 SNP loci from the coral linkage map selected from Wang** ***et al.*** **[27].**
Additional file 5:
**Standard deviations of differences between estimated and expected allele frequencies across melting temperature range.** The standard deviation (i.e. inaccuracy) increased towards either end of the melting temperatures. The smallest standard deviation was observed at the temperatures between 64.4-64.5°C, indicating the highest accuracy in estimating allele frequencies at this temperature range. Consistent with this, the largest difference in fluorescence level between two homozygotes was also observed within the same temperature range for C70S236 marker. Additional file 6:
**qHRM detection of novel alleles and possible gene duplication event.** The probe peaks for SNP C1023S218 show two individuals heterozygous for different alleles and the presence of a third allele within a single individual. Three individuals of the six genotyped for this locus showed the same pattern of three alleles each. Additional file 7:
**Outlier sample identified from Wilkie population using the SNP marker C70S236.** The outlier sample has two peaks with low-melting peak being higher than the other. A heterozygous (Hetero) and two homozygous (Homo high and Homo low) were clearly different from the outlier, indicating that the outlier sample may cause an error in allele frequency estimates for Wilkie population. Additional file 8:
**Replicate errors associated with 25 populations and 5 SNP markers.**
Additional file 9:
**Melt curves of three reference samples.** Two homozygotes and one heterozygote show the same pattern of normalized fluorescence level among markers with different combinations of nucleotides at SNP sites.

## Abbreviations

HRM: High resolution melting
qHRM: Quantitative high resolution melting
qPCR: Quantitative PCR
SNP: Single nucleotide polymorphism
