# Supplementary figures and images for: Quantitative high resolution melting: two methods to determine SNP allele frequencies from pooled samples
Source: BMC Genet. 2015 Jun 13;16:62. doi: 10.1186/s12863-015-0222-z (PMC4465018; doi:10.1186/s12863-015-0222-z)

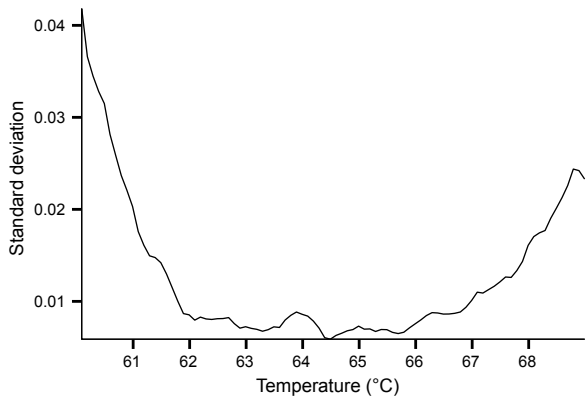

Supplement: Additional file 5: — Standard deviations of differences between estimated and expected allele frequencies across melting temperature range. The standard deviation (i.e. inaccuracy) increased towards either end of the melting temperatures. The smallest standard deviation was observed at the temperatures between 64.4-64.5°C, indicating the highest accuracy in estimating allele frequencies at this temperature range. Consistent with this, the largest difference in fluorescence level between two homozygotes was also observed within the same temperature range for C70S236 marker. [file 12863_2015_222_MOESM5_ESM.pdf]

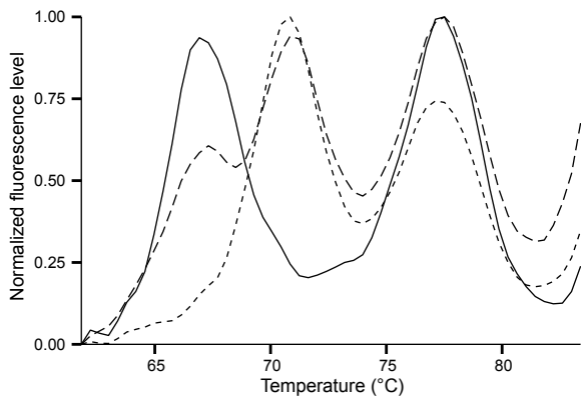

Supplement: Additional file 6: — qHRM detection of novel alleles and possible gene duplication event. The probe peaks for SNP C1023S218 show two individuals heterozygous for different alleles and the presence of a third allele within a single individual. Three individuals of the six genotyped for this locus showed the same pattern of three alleles each. [file 12863_2015_222_MOESM6_ESM.pdf]

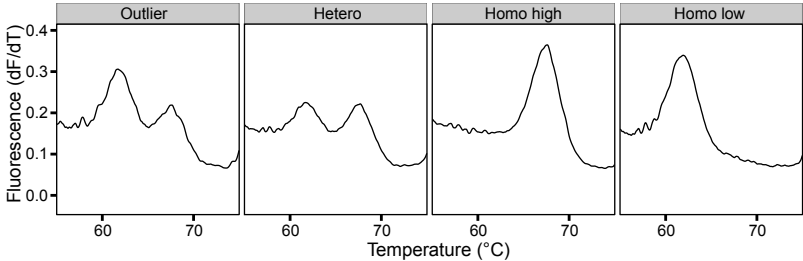

Supplement: Additional file 7: — Outlier sample identified from Wilkie population using the SNP marker C70S236. The outlier sample has two peaks with low-melting peak being higher than the other. A heterozygous (Hetero) and two homozygous (Homo high and Homo low) were clearly different from the outlier, indicating that the outlier sample may cause an error in allele frequency estimates for Wilkie population. [file 12863_2015_222_MOESM7_ESM.pdf]

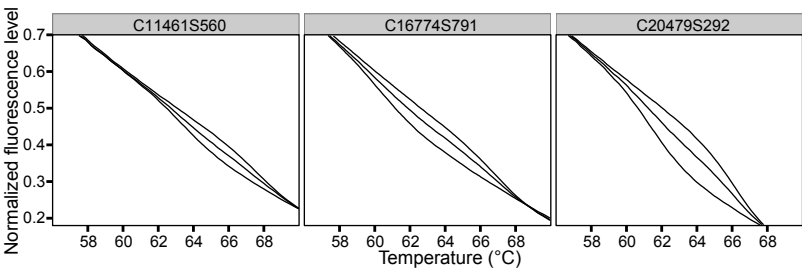

Supplement: Additional file 9: — Melt curves of three reference samples. Two homozygotes and one heterozygote show the same pattern of normalized fluorescence level among markers with different combinations of nucleotides at SNP sites. [file 12863_2015_222_MOESM9_ESM.pdf]
